# Optimization of border irrigation variables based on a correction factor for irrigation quota

**DOI:** 10.1016/j.heliyon.2024.e40116

**Published:** 2024-11-05

**Authors:** Mohamed Khaled Salahou, Xiaoyuan Chen, Yupeng Zhang, Haishen Lü, Xiyun Jiao

**Affiliations:** a School of Biology and Agriculture, Shaoguan University, Shaoguan 512005, China; b Northern Guangdong Soil Environment Observation and Research Station, Shaoguan University, Shaoguan 512005, China; c Engineering Technology Research Center for Efficient Utilization of Water and Land Resources in North Guangdong, Shaoguan University, Shaoguan 512005, China; d State Key Laboratory of Hydrology-Water Resources and Hydraulic Engineering, Hohai University, Nanjing 210098, China

**Keywords:** Irrigation, Cutoff time, Infiltration, Yield, Optimization, Irrigation efficiency

## Abstract

The optimization of surface irrigation variables, i.e., the selection of the optimal combination of the inflow rate per unit width (q) and cutoff time (tco), is essential for obtaining high performance. The main objective of this study was to optimize irrigation variables by considering different irrigation requirements and the total loss model, which includes the irrigation water loss and crop yield loss. A correction factor for the irrigation quota (C_f_) was introduced to achieve this objective. C_f_ considers different irrigation requirements, as it represents the ratio of the actual irrigation quota for certain irrigation to the designed irrigation quota (D_req_). Uniform design theory was used to determine random combinations of q and C_f_ from a selected range. The q value ranged from 3 to 9 L m^−1^. s^−1^. Because the actual irrigation quota does not greatly deviate from the design irrigation quota, C_f_ should reach approximately 1.00. The selected C_f_ ranged from 0.80 to 1.38. To obtain higher crop yields, lower economic losses, and more reasonable design variables for surface irrigation, a total loss model for uneven border irrigation was established, which was defined as the objective function to optimize border irrigation design—the total loss model was combined with uniform design theory and the WinSRFR model to analyze different scenarios. The results showed that C_f_ has a clear meaning and exerts a favorable application effect on the irrigation performance evaluation indicators and can be used to design border irrigation systems. Based on the total loss model for uneven border irrigation, the optimal irrigation variables q and C_f_ for design irrigation quotas of 60, 80, and 100 mm are q = 6.22 L m^−1^. s^−1^ and C_f_ = 1.17; q = 4.60 L m^−1^. s^−1^ and C_f_ = 1.14; and q = 3.80 L m^−1^. s^−1^ and C_f_ = 1.10, respectively. Compared with the conventional design results, q in the optimal design results based on the loss model decreased, C_f_ increased, and the total loss significantly decreased. Optimization of irrigation variables based on the border irrigation loss model could ensure favorable irrigation performance evaluation indicators, improve the water use efficiency, provide higher crop yields, and minimize the total economic losses caused by uneven irrigation.

## Highlights

• A correction factor for the irrigation quota (C_f_) was introduced.
• A total loss model for uneven border irrigation was established and defined as the objective function to optimize the irrigation design.
• Uniform design theory was used to determine random combinations of the inflow rate per unit width and C_f_.

## 1 Introduction

The border irrigation system is widely adopted for wheat production in the North China Plain because of its low energy and cost requirements [[1], [2], [3], [4]].

Many factors affect the performance of border irrigation, including soil infiltration characteristics, roughness coefficient, field slope, border length (L), border width (B), inflow rate per unit width (q), and cutoff time (irrigation time, tco) [[5], [6], [7], [8], [9], [10]]. These variables can be divided into controllable and uncontrollable variables, according to the difficulty of control [11]. The fixed variables are soil infiltration characteristics, roughness coefficient, and field slope. The border length and width are design factors that are difficult to modify and are usually specified by farmers [1,11]. Chen, Ouyang, Sun, Wu and Li [12] optimized border dimensions using a simulation model, household surveys, and field measurements, but merging small plots into large ones is difficult due to different land-use rights. However, q and tco are the essential variables for surface irrigation design and management [1,[13], [14], [15], [16], [17], [18]], which can vary between irrigation events and hence can be used to improve the irrigation system performance.

When the field size (L and B) and crop planting type in the experimental area are known, variables such as soil infiltration parameters, longitudinal slope of the field, and roughness coefficient, which are uncontrollable variables, can be directly measured or estimated and inverted through field experiments [1,[19], [20], [21], [22], [23]]. Notably, q and tco are controllable variables, and their combination can be designed based on other border irrigation variables [13,14,17,[24], [25], [26]]. The design of border irrigation variables entails combination design of management or controllable variables based on the known geometric parameters of the border system and soil infiltration characteristics. Hence, conventional design of border irrigation variables involves field experiments or computer simulations of a combination of controllable variables (q and tco) at different levels to maximize border irrigation performance indicators, after which the best combination can be selected to guide field irrigation [1,14,27]. To avoid problems, i.e., the predicted advance is sensitive to slight variations in the inflow rate, roughness coefficient, and infiltration parameters, Bautista, Clemmens, Strelkoff and Niblack [28] replaced the q–tco combination by the ratio R, which is defined as the ratio between tco and the final advance time in cases where tco follows the final advance and as the advance distance at tco divided by the field length in cases where tco precedes the last advance. As the ratio R does not consider different irrigation quotas, the irrigation quota correction coefficient (the ratio of the corrected irrigation quota to the design irrigation quota) (C_f_) was proposed and applied to border irrigation design in this study with the selected irrigation performance evaluation indicators as the optimization goal. The use of the irrigation quota correction coefficient can aid in the optimization of crop growth and development. This is achieved by adjusting the water supply based on crop type, soil type, and weather conditions. This approach reduces water wastage and ensures crops receive the appropriate water required.

The border irrigation method also suffers the problem of an uneven distribution of infiltration water along the length of the border [29]. After a specific irrigation session, the infiltration water depth in some fields does not reach the designed irrigation quota D_req_. In contrast, the infiltration water depth in another part of the field can exceed the design irrigation quota D_req_. The underirrigated part cannot meet the crop water requirements, causing crop yield losses. The overirrigated part is prone to deep percolation, resulting in irrigation water loss and increased irrigation management costs. The optimal design of surface irrigation can be obtained by studying the reasonable combination of irrigation variables under complete and proper utilization of limited water resources so that the applied irrigation water can meet the crop water needs. To obtain higher crop yields, lower economic losses, and more reasonable design variables for border irrigation, a total loss model was established for uneven border irrigation, which was adopted as the objective function to optimize the design of uneven border irrigation. The total loss model was combined with uniform design theory [30,31] and the WinSRFR model [28,32,33] to determine random combinations of q and C_f_ from a selected range, and analyze the obtained scenarios.

Considering irrigation water and crop yield loss in the overall loss model provides a more comprehensive approach to designing surface irrigation systems. Hence, the objectives of this study were as follows: (1) to consider different irrigation requirements to optimize irrigation variables by proposing and applying the irrigation quota correction coefficient C_f_ in border irrigation design, (2) to investigate the impact of C_f_ on the irrigation performance evaluation indicators and the total loss model, and (3) to optimize the border irrigation variables based on the total loss model, which includes the irrigation water loss and crop yield loss.

## 2 Material and methods

### 2.1 Study area

In this study, experiments involving 27 border fields [1] were conducted at the CAS Ecological Agricultural Experiment Station in the town of Nanpi in Hebei Province, China, at longitude, latitude, and elevation of 116°40′E, 38°06′N and 20 m, respectively. This area is located in a monsoon climate zone with an annual evaporation range between 1500 and 1800 mm. The mean annual precipitation and temperature at the study site are approximately 567.4 mm and 12.3 °C, respectively. Rainfall generally occurs during the summer, with 73 %, 11 %, 13 %, and 3 % of the annual precipitation occurring during the summer, spring, autumn, and winter, respectively. The soil at the site is classified as silt loam (67.02 % silt, 25.19 % sand, and 7.79 % clay on average), and the groundwater depth is approximately 4 m. The volumetric soil water content at field capacity before irrigation ranged from 15.8 % to 19.6 %. The bulk densities were 1.21 g cm^−3^ in the surface soil layer (0–5 cm), 1.51 g cm^−3^ in the subsurface soil layer (60–80 cm), and 1.45 g cm^−3^ at a depth of 1 m.

### 2.2 Irrigation design variables

The field slope differs between the borders, with an average of 0.0022. The uncontrollable variables (i.e., L and B) were similar to the border dimensions used by local farmers (L = 100 m, B = 3.7 m). The Kostiakov equation, i.e., $Z=k\tau^{\alpha }$, where Z is the cumulative infiltration depth in m, τ is the intake opportunity time in min, and k is a coefficient with units of mm/min^-a^, was used in the analysis process to determine the infiltration function. The values of the infiltration parameters (k and a) and roughness coefficient of the evaluated field were established with the WinSRFR hydraulic simulation model (Bautista et al., 2009a, 2009b; Bautista and Schlegel, 2017b; Bautista et al., 2015) and a trial-and-error method [1]. Salahou, Jiao and Lu [1] published the details of the calculation process for the tested borders, values of the Kostiakov equation, and roughness coefficient. It is worth noting that different trials were conducted using of Modified Kostiakov equation ($Z=k\tau^{\alpha }+b\tau +c$) and in each case, the values of the extra parameter b (the final or steady infiltration rate) and c (the instantaneous infiltration depth into soil macropores) were close to zero, which does not indicate strong evidence of macrospore flow.

In the interest of brevity, we calculated the average soil infiltration parameters of the 27 tested borders. However, the average soil infiltration parameters are approximately equal to the parameters of border No. 14 [1]. Hence, in this work, the soil infiltration parameters and roughness coefficient of border No. 14 ($Z=122.24\tau^{0.68},slope=0.0025,and\;roughness\;coefficient=0.06$) were used in the analysis.

### 2.3 Correction factor for the irrigation quota

The main objective was to satisfy the design irrigation quota and meet the crop water needs. Hence, the irrigation quota correction coefficient (C_f_) was proposed instead of the irrigation time (tco) to design the border irrigation variables. Since surface irrigation is a prerequisite for meeting the crop water needs, the irrigation design variables restrict the actual irrigation quota and fluctuate around the designed irrigation quota. Hence, the irrigation quota correction coefficient can be defined as follows:

(1) $$C_{f}=\frac{m}{D_{req}}$$

where C_f_ is the irrigation quota correction coefficient, dimensionless; m is the actual irrigation quota for certain irrigation, mm; and D_req_ is the design irrigation quota (infiltration depth), mm, which is determined based on the measured soil moisture content before irrigation in the field or irrigation experience of local farmers, the designed irrigation depth of crops and the field water capacity.

Notably, tco is affected by m, L, and q and can be calculated as follows:

(2) $$t_{co}=\frac{mL}{60q}=\frac{C_{f}D_{req}L}{60q}$$

Eq. (2) reveals that when D_req_, q, and the border field size (L and W) are the same, C_f_ exhibits a positive linear relationship with tco; notably, with increasing C_f_, tco increases. D_req_ is affected by the crop type, rainfall, and temperature. The winter wheat is irrigated two times per season in the study area (pre-sowing and spring irrigation), the experiment was conducted during the spring irrigation event, and the required application water depth was 60 mm [34]. As part of the calculation process, water depths of 80 mm and 100 mm were also considered for compression and expansion purposes, respectively.

In this work, q and C_f_ were selected as controllable factors to design the border irrigation system. The recommended inflow rate range for achieving high irrigation performance, as suggested by Morris, Hussain, Gillies and O'Halloran [24] and Salahou, Jiao and Lu [1], is between 2 and 7 L s^−1^ m^−1^ and 2.81 and 6.91 L s^−1^ m^−1^, respectively. This study will use the same border experiments as Salahou, Jiao and Lu [1], and the selected q values will vary between 3 and 9 L s^−1^ m^−1^. Because the actual irrigation quota does not greatly deviate from the designed irrigation quota, C_f_ should reach approximately 1.00. The selected C_f_ values ranged from 0.80 to 1.38. The step sizes of q and C_f_ were set to 0.2 and 0.02, respectively, with 30 levels.

### 2.4 Uniform design theory

Uniform design (UD) theory was proposed by Refs. [30,[35], [36], [37]]. UD is based on the quasi-Monte Carlo method or number theoretic method [35]. used so-called good lattice point (glp) sets to construct many tables for UD. UD tables can be denoted as follows:

(3) $$U_{n}\left(q^{s}\right)$$

where U denotes UD, n is the number of runs, s is the number of factors, and q is the number of levels. UD tables can be obtained from the Hong Kong Baptist University Mathematical Department website (http://www.math.hkbu.edu.hk/UniformDesign/).

The UD method was developed to select a small but representative set of points from a given set of points such that the sampled points are uniformly distributed. Salahou, Chen, Zhang, Jiao, Lü and Shah [31] introduce the UD method as a parameter sampling method. In the following section, UD theory is used to select random combinations of q and C_f_ from a determined range.

The combinations of q and C_f_ obtained by UD theory with the uncontrollable variables ($k=122.24\;mm.\min^{-1},\propto =0.68,slope=0.0025,and\;roughness\;coefficient=0.06$), established in Section 2.1, were fed into the WinSRFR model to evaluate different scenarios.

### 2.5 Evaluation irrigation system

#### 2.5.1 Irrigation performance indicators

The evaluation indicators of surface irrigation include the application efficiency (AE), distribution uniformity (DU), and requirement efficiency (RE) (their mathematical expressions have been defined in many studies, e.g., Refs. [1,38,39]), which reflect the degree to which field irrigation water is effectively used by crops after irrigation, the degree of uniformity of the distribution and the degree of satisfaction of the crop water requirements, respectively. The AE, RE and DU indicators were defined as follows:

(4) $$AE=\frac{average\;depth\;of\;infiltrated\;water\;stored\;in\;the\;root\;zone\;}{depth\;of\;total\;water\;applied}$$

(5) $$RE=\frac{average\;depth\;of\;infiltrated\;water\;stored\;in\;the\;root\;zone\;}{water\;required\;in\;the\;root\;zone\;}$$

(6) $$DU=\frac{average\;of\;the\;lowest\;25\%\;of\;infiltration\;depths\;}{average\;infiltration\;depth\;in\;the\;whole\;field}$$

To better explain the irrigation performance, a comprehensive evaluation indicator Y was used in final evaluation [19]. Y exhibits a maximum value of 100 %, and the larger Y is, the higher the irrigation performance.

(7) $$Y=\sqrt[{3}]{AE\times RE\times DU}$$

where Y is the comprehensive performance indicator of surface irrigation.

#### 2.5.2 Total loss model of border irrigation

As previously mentioned, the total loss model includes the irrigation water loss and crop yield loss, which can be defined as follows.

A. Crop yield loss

Assuming that the amount of insufficient irrigation water in the border field during the crop growth period is as follows [40]:

(8) $${\Delta ET}_{i}^{\prime }=\left\{ \begin{array}{c} {ET}_{m}-{ET}_{ai}\;{ET}_{m}\geq {ET}_{ai}\\ 0\;\;\;\;\;\;\;\;\;\;\;\;\;\;\;{\;ET}_{m}<\;{ET}_{ai}\; \end{array}\right.$$

where ${\Delta ET}_{i}^{\prime }$ is the amount of insufficient irrigation water in the border field during the crop growth period, mm; ${ET}_{m}$ is the maximum evapotranspiration amount during the crop growth period, mm; and ${ET}_{ai}$ is the actual evapotranspiration amount in the border field during the entire crop growth period, mm.

If only the influence of uneven irrigation at a certain time is considered while ignoring the influence of other irrigation and rainfall, Eq. (8) can be simplified as:

(9) $${\Delta ET}_{i}^{'}=\left\{ \begin{array}{c} D_{req}-m_{i}\;D_{req}\geq m_{i}\\ 0\;\;\;\;\;\;\;\;\;\;\;\;\;\;\;D_{req}<\;m_{i}\; \end{array}\right.$$

where $D_{req}$ is the design irrigation quota for certain crop irrigation, mm, and $m_{i}$ is the actual infiltration water amount at the border, mm.

The crop yield loss caused by a lack of irrigation [[40], [41], [42], [43]] can be obtained as follows:

(10) $$\left(1-\frac{Y_{ai}}{Y_{m}}\right)=K_{y}\left(1-\frac{{ET}_{ai}}{{ET}_{m}}\right)$$

where $Y_{ai}$ is the actual harvested yield, kg.hm^−2^; $Y_{m}$ is the maximum harvested yield under sufficient irrigation conditions, kg.hm^−2^; and $K_{y}$ is the crop response coefficient or sensitivity coefficient, dimensionless.

Substituting Eq. (9) into Eq. (10), we can obtain the crop yield loss due to uneven irrigation (underirrigation) in the field.

(11) $$\Delta Y_{i}=\left\{ \begin{array}{c} Y_{m}\times K_{y}\frac{D_{req}-m_{i}\;}{{ET}_{m}}\;D_{req}\geq m_{i}\\ 0\;\;\;\;\;\;\;\;\;\;\;\;\;\;\;D_{req}\leq m_{i}\; \end{array}\right.$$

where $\Delta Y_{i}$ is the yield loss due to underirrigation in the field, kg.hm^−2^; and the other variables are as previously defined.

The total crop yield loss caused by underirrigation per unit area of borderland is:

(12) $$S=\frac{1}{L}\int_{0}^{L}\Delta Y_{i}dx$$

where S is the crop yield loss due to underirrigation per hectare, kg.hm^−2^; and x is the distance along the border length direction on, m.

B. Irrigation volume loss

Overirrigation can lead to a waste of water resources and increased management costs. Similar to underirrigation, if only the loss of the irrigation volume due to overirrigation at a particular time of irrigation is considered, the volume of overirrigation at the border can then be expressed as:

(13) $${\Delta ET}_{i}^{\prime \prime }=\left\{ \begin{array}{c} m_{i}-D_{req}\;m_{i}\geq D_{req}\\ 0\;\;\;\;\;\;\;\;\;\;\;\;\;\;\;m_{i}<\;D_{req}\; \end{array}\right.$$

where ${\Delta ET}_{i}^{\prime \prime }$ is the amount of overirrigation in the field after a certain irrigation, mm.

The total water loss caused by uneven irrigation (overirrigation) per unit area of border is:

(14) $$W=10\frac{1}{L}\int_{0}^{L}{\Delta ET}_{i}^{\prime \prime }dx$$

where W is the amount of irrigation water loss due to overirrigation per hectare, m^3^.hm^−2^; x is the distance along the border length direction, m; and the other variables are as previously defined.

C. Total loss model

Loss functions of uneven irrigation were established from the perspective of the economic value. The crop yield loss per hectare of the border field due to underirrigation is S. If the crop price is P, the economic value of the crop yield loss can be calculated as follows:

(15) $$L_{1}=S\times P$$

where L_1_ is the economic loss caused by the crop yield loss per hectare, Yuan.hm^−2^; and P is the crop price, Yuan.kg^−1^.

For typical well irrigation areas in the North China Plain, the main source of irrigation water is extracted groundwater. The waste of water resources and increased management costs due to the loss of irrigation water are reflected in the expenditure of irrigation water fees. If the water fee in the well irrigation area in the North China Plain is Q (including electricity and management fees), the economic value of the loss of irrigation water is:

(16) $$L_{2}=W\times Q$$

where L_2_ is the economic loss caused by the loss of irrigation water per hectare of the border field, Yuan.hm^−2^; and Q is the water fee in the well irrigation area in the North China Plain, Yuan.hm^−3^.

Therefore, border fields are either under- or overirrigated, resulting in both crop yield loss and irrigation water loss. Hence, the total loss (L_T_) caused by uneven irrigation is:

(17) $$L_{T}=L_{1}\times L_{2}$$

In typical irrigation design, uncontrollable variables (k, a, slope, and roughness coefficient) are determined and maintained unchanged, and the controllable variables (q, and tco) are the main variables affecting L_T_. The total loss model provides new ideas and methods for the design of irrigation variables.

The parameters of the total loss L_T_ include the parameters of Eq. (10), crop price, and water fees in the experimental area. The parameters of Eq. (10), which is the winter wheat moisture production function for Hebei Province, are K_y_ = 1.2617, Y_m_ = 6615 kg hm^−2^, and ET_m_ = 396.31 mm [42]. According to the National Development and Reform Commission, the wheat purchase price was 2.34 yuan.kg^−1^ in 2023 (https://zfxxgk.ndrc.gov.cn/web/iteminfo.jsp?id=18959). Several factors affect the water cost, such as the groundwater level, management fee, and electricity fee, making it difficult to determine, and the value differs from one area to another [42]. Due to reduced river seepage and increased groundwater extraction, the maximum depth of shallow groundwater levels in the North China Plain has reached 65 m [44,45]. The decline in the groundwater level affects the water fee [46]. In this study, the calculated water fee (including management and electricity fees) in a typical well irrigation area in the North China Plain is 0.8 yuan.m^−3^ [46].

### 2.6 Statistical analysis

There are many methods for optimizing the obtained uniform design results [47]. Multiple linear regression analysis was used to optimize the designed irrigation variables [48,49]. Hence, the different combinations of q and C_f_ were evaluated using the multiple linear regression model (2 s-order predictor variables (Eq. (18)) and two third-order predictor variables (Eq. (19))) as follows:

(18) $$Z=\beta_{0}+\beta_{1}q+\beta_{2}C_{f}+\beta_{3}q^{2}+\beta_{4}{C_{f}}^{2}+\beta_{5}qC_{f}$$

where Z is the outcome (comprehensive evaluation indicator Y, or L_T_ in the last section); $\beta_{0}$ is the model intercept; $\beta_{1}$, $\beta_{2}$, $\beta_{3}$, $\beta_{4}$, and $\beta_{5}$ are model coefficients; q and C_f_ are the model covariates; $\beta_{0}+\beta_{1}q+\beta_{2}C_{f}$ denotes the linear component; $\beta_{3}q^{2}+\beta_{4}{C_{f}}^{2}$ denotes the quadratic component; and $\beta_{5}qC_{f}$ is the cross product or interaction component.

(19) $$Z=\beta_{0}+\beta_{1}q+\beta_{2}C_{f}+\beta_{3}q^{2}+\beta_{4}q^{3}+\beta_{5}{C_{f}}^{3}+\beta_{6}q{C_{f}}^{2}$$

The regression equation results were analyzed using the backward elimination method [48,50] in SPSS statistical software (IBM-SPSS, 19, USA). The backward elimination process begins by fitting a multiple linear regression model with all the independent variables [50]. The variable with the highest p value is removed from the model, and a new model is determined. This process is repeated until all variables in the model yield a p value below a certain threshold, typically 0.05.

The genetic algorithm (GA) [51] is a well-known algorithm that can be used to determine the maximum value (Z) of the final multiple linear regression model equation (Eqs. (18), (19)). The GA is a method for solving both constrained and unconstrained optimization problems that is based on natural selection, the process that drives biological evolution [52]. The GA aims to repeatedly modify a population of individual solutions. Microsoft Excel 2021 software was used to implement the GA through Microsoft Excel Solver.

## 3 Results and discussion

### 3.1 Conventional design method for irrigation variables

The analysis results for a design irrigation quota of 60 mm are shown in detail (the results for D_req_ = 80 and 100 are shown in brief). Considering irrigation performance indicator Y as the evaluation target, conventional design of the border irrigation variables was performed. Table 1 provides the q and C_f_ values.

**Table 1:** Range of the inflow rate and correction factor values.

Level	q L.s^−1^.m^−1^	C_f_	Level	q L.s^−1^.m^−1^	C_f_
1	3.0	0.80	16	6.0	1.10
2	3.2	0.82	17	6.2	1.12
3	3.4	0.84	18	6.4	1.14
4	3.6	0.86	19	6.6	1.16
5	3.8	0.88	20	6.8	1.18
6	4.0	0.90	21	7.0	1.20
7	4.2	0.92	22	7.2	1.22
8	4.4	0.94	23	7.4	1.24
9	4.6	0.96	24	7.6	1.26
10	4.8	0.98	25	7.8	1.28
11	5.0	1.00	26	8.0	1.30
12	5.2	1.02	27	8.2	1.32
13	5.4	1.04	28	8.4	1.34
14	5.6	1.06	29	8.6	1.36
15	5.8	1.08	30	8.8	1.38

With the use of DU theory, we generated random combinations of q and C_f_ on the basis of Table 1, where n = 30 and s = 2. Hence, uniform design table U_30_ (30^2^) was used for scheme design. Table 2 shows the design levels of q and C_f_.

**Table 2:** Combinations of q and C_f_ based on U_30_ (30^2^).

Level	q L.s^−1^.m^−1^	C_f_	Level	q L.s^−1^.m^−1^	C_f_
1	3.0	0.80	16	6.0	1.10
2	3.2	0.82	17	6.2	1.12
3	3.4	0.84	18	6.4	1.14
4	3.6	0.86	19	6.6	1.16
5	3.8	0.88	20	6.8	1.18
6	4.0	0.90	21	7.0	1.20
7	4.2	0.92	22	7.2	1.22
8	4.4	0.94	23	7.4	1.24
9	4.6	0.96	24	7.6	1.26
10	4.8	0.98	25	7.8	1.28
11	5.0	1.00	26	8.0	1.30
12	5.2	1.02	27	8.2	1.32
13	5.4	1.04	28	8.4	1.34
14	5.6	1.06	29	8.6	1.36
15	5.8	1.08	30	8.8	1.38

In addition to the uncontrollable variables (k = 122.24 mm.min^-a^, a = 0.68, slope = 0.0025, and roughness coefficient = 0.06) (see Section 2.3)), the flow rate Q and tco are input parameters in the WinSRFR model. Under a known border size (L = 100 m, and B = 3.7 m) and design irrigation quota D_req_ (D_req_ = 60 mm, as an example), the flow rate into the border is Q = q.B, and the irrigation time is tco = C_f_.L.D_req_/(60q).

Based on Table 2, the WinSRFR5.1 model was used to simulate different levels, and AE, DU, RE, and Y at each level were obtained. The simulation results are listed in Table 3.

**Table 3:** Conventional design results for the irrigation indicators (D_req_ = 60 mm).

Level	q L.s^−1^.m^−1^	C_f_	Q L.s^−1^	tco min	AE %	DU%	RE%	Y%
1	7.6	1.28	28.1	16.8	0.78	0.82	1.00	0.862
2	7.4	0.90	27.4	12.2	0.99	0.89	0.89	0.922
3	3.0	1.02	11.1	34.0	0.75	0.12	0.77	0.411
4	4.6	1.14	17.0	24.8	0.86	0.80	0.98	0.877
5	3.8	1.10	14.1	28.9	0.81	0.50	0.89	0.712
6	5.0	0.92	18.5	18.4	0.96	0.65	0.88	0.819
7	6.6	0.86	24.4	13.0	1.00	0.85	0.86	0.901
8	4.0	0.96	14.8	24.0	0.88	0.42	0.84	0.677
9	7.0	1.00	25.9	14.3	0.96	0.89	0.96	0.936
10	3.4	0.84	12.6	24.7	0.87	0.10	0.73	0.399
11	5.2	1.06	19.2	20.4	0.92	0.84	0.97	0.908
12	5.8	1.18	21.5	20.3	0.85	0.89	1.00	0.911
13	6.8	1.38	25.2	20.3	0.72	0.83	1.00	0.842
14	6.4	1.08	23.7	16.9	0.92	0.89	0.99	0.932
15	6.2	1.26	22.9	20.3	0.80	0.88	1.00	0.890
16	8.0	0.82	29.6	10.3	1.00	0.89	0.82	0.900
17	4.2	1.36	15.5	32.4	0.74	0.85	1.00	0.857
18	3.6	1.20	13.3	33.3	0.75	0.52	0.91	0.708
19	8.4	1.04	31.1	12.4	0.91	0.86	0.95	0.906
20	8.2	1.34	30.3	16.3	0.75	0.78	1.00	0.836
21	7.8	1.12	28.9	14.4	0.88	0.85	0.99	0.905
22	5.4	1.32	20.0	24.4	0.76	0.89	1.00	0.878
23	5.6	0.80	20.7	14.3	1.00	0.60	0.80	0.783
24	8.6	0.94	31.8	10.9	0.95	0.88	0.89	0.906
25	7.2	1.16	26.6	16.1	0.86	0.86	1.00	0.904
26	4.4	0.88	16.3	20.0	0.94	0.44	0.83	0.700
27	3.2	1.30	11.8	40.6	0.68	0.41	0.88	0.626
28	6.0	0.98	22.2	16.3	0.98	0.87	0.96	0.935
29	8.8	1.22	32.6	13.9	0.81	0.79	0.99	0.859
30	4.8	1.24	17.8	25.8	0.81	0.88	1.00	0.893

There are 12 levels with Y values greater than 0.900, Q values between 19.2 and 31.8 L s^−1^, and tco values between 10.3 and 20.4 min; notably, q varies between 5.2 and 8.6 L s^−1^ m^−1^, and C_f_ varies between 0.82 and 1.18, which does not reach the boundary value of the selected interval (0.8–1.38). In addition, with increasing q, C_f_ shows a downward trend, indicating that under low-flow conditions, a longer irrigation time is needed to achieve better irrigation effects. Conversely, the opposite is true under high-flow conditions.

The significance test results for the above conventional design of the second-order regression equation are provided in Table 4, and the coefficients of the second-order regression equation are shown in Table 5. The adjusted R^2^ value of the second-order regression equation is 0.929, the fitting effect is favorable, and the standard estimation error is small. The significance value of the F test is far less than 0.001, and the second-order regression equation is extremely significantly related. Moreover, Table 5 reveals that the significance value of the coefficient *t*-test of the regression equation meets the highly significant correlation requirements.

**Table 4:** Significance test of the second-order regression equation (D_req_ = 60 mm).

Multiple R	R^2^	Adjusted R^2^	Standard Error	*F*	*Significance F*
0.970	0.941	0.929	0.929	77.076	5.5E-14

**Table 5:** Second-order regression equation coefficients and test values (D_req_ = 60 mm).

Variable Name	Coefficients	Standard Error	*T Stat*	*P Value*
Intercept	−2.6438	0.3489	−7.58	8.13E-08
q L.s^−1^.m^−1^	0.5631	0.0394	14.31	3.02E-13
C_f_	3.0772	0.5745	5.36	1.68E-05
q^2^	−0.0284	0.0026	−11.09	6.23E-11
C_f_^2^	−0.9202	0.2561	−3.59	0.00146
qXC_f_	−0.1581	0.0235	−6.73	5.78E-07

The GA is used to obtain the maximum value of the second-order regression equation (i.e., $Y=-2.6438+0.5631q+3.0772C_{f}-0.0284q^{2}-0.9202C_{f}^{2}-0.1581qC_{f}$), and the conventional design for a design irrigation quota of 60 mm is optimized, with q = 6.9 L s^−1^ m^−1^ and C_f_ = 1.08.

The significance test results for the conventional design of the third-order regression equation are listed in Table 6, and the coefficients of the third-order regression equation are shown in Table 7. The adjusted R^2^ value of the third-order regression equation is 0.976, the fitting effect is excellent, and the standard estimation error is small. The significance value of the F test is much less than 0.001, and the third-order regression equation is highly significantly correlated. The third-order regression equation provides better fitting results than the second-order regression equation. In addition, Table 7 indicates that the significance value of the coefficient *t*-test of the third-order regression equation meets the highly significant correlation requirements.

**Table 6:** Significance test of the third-order regression equation (D_req_ = 60 mm).

Multiple R	R^2^	Adjusted R^2^	Standard Error	*F*	*Significance F*
0.990	0.981	0.976	0.022	196.62	1.41E-18

**Table 7:** Third-order regression equation coefficients and test values (D_req_ = 60 mm).

Variable Name	Coefficients	Standard Error	*T Stat*	*P Value*
Intercept	−3.091	0.218	−14.159	7.6E-13
q L.s^−1^.m^−1^	1.155	0.100	11.605	4.3E-11
C_f_	1.538	0.171	8.997	5.4E-09
q^2^	−0.149	0.018	−8.522	1.4E-08
q^3^	0.007	0.001	6.923	4.67E-07
C_f_^3^	−0.129	0.047	−2.728	0.012
qXC_f_^2^	−0.073	0.006	−11.605	4.3E-11

The GA is used to determine the maximum value of the third-order regression equation, and the conventional design for a design irrigation quota of 60 mm is optimized, with q = 6.l0 L.s^−1^.m^−1^ and C_f_ = 1.15.

Notably, the design results of the second- and third-order regression equations differ, and the difference is significant. The results of both equations were fed into the WinSRFR5.1 model for simulation and compared with the simulation results of the uniform design scheme (Table 3)with the maximum comprehensive index Y as the goal. D_req_ was set to 60 mm. The optimal results for the irrigation variables are shown in Table 8. Although the test values of the second- and third-order regression equations are significant, the optimization result does not encompass the optimal irrigation variables.

**Table 8:** Selection of the best solution for the conventional design (D_req_ = 60 mm).

Method	q L.s^−1^.m^−1^	C_f_	AE	DU	RE	Y
Level 9	7.00	1.00	0.96	0.89	0.96	0.936
Second Order	6.91	1.08	0.92	0.88	0.99	0.929
Third Order	6.10	1.15	0.87	0.89	1.00	0.918

Comparing the design in Table 8, the design with the most significant comprehensive index Y was selected as the best design, i.e., level 9 (q = 7.0 L s^−1^ m^−1^, and C_f_ = 1.00).

The design results at the three typical D_req_ levels (60, 80, and 100 mm) are listed in Table 9. With increasing D_req_, q gradually decreases, and C_f_ gradually increases. Under the combined values of q and C_f_, tco increases. The conventional design scheme demonstrates that C_f_ varies between 1 and 1.10; notably, the actual irrigation quota slightly differs from the design D_req_ value (the irrigation volume is slightly different), which improves the degree of the applied irrigation meeting the crop water needs. RE is significantly higher than the other two evaluation indicators.

**Table 9:** Conventional design results for the different water requirement quotas.

D_req_ mm	q L.s^−1^.m^−1^	C_f_	AE	DU	RE	Y
60	7.0	1.00	0.96	0.89	0.96	0.936
80	5.2	1.06	0.93	0.89	0.99	0.936
100	3.8	1.10	0.91	0.91	1.00	0.939

### 3.2 Effect of C_f_ on the irrigation performance indicators

To study the impact of C_f_ on the irrigation evaluation indicators and ensure that the research goals have been achieved, D_req_ and the optimal q values were analyzed. The specific design is summarized in Table 10.

**Table 10:** Scenarios to study the impact of the irrigation quota correction coefficient C_f_ on the irrigation evaluation indicators.

D_req_ mm	q L.s^−1^.m^−1^	C_f_	
		Value	Step
60	7.0	0.80–1.40	0.05
80	5.2		
100	3.8		

**Fig. 1:**
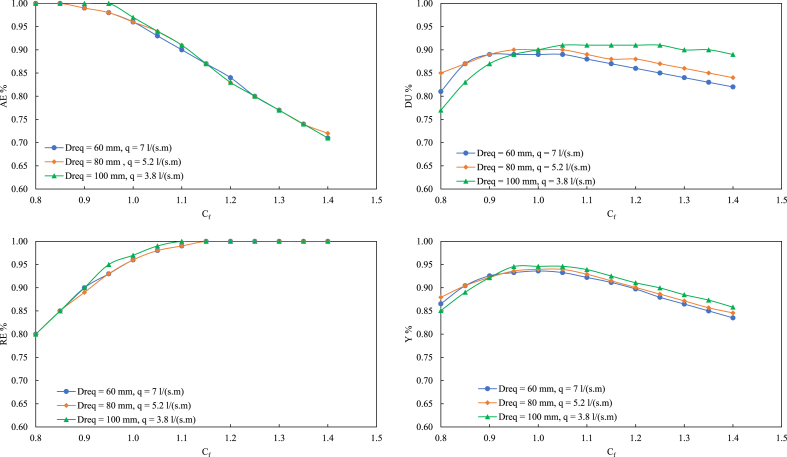
Influence of Cf on the irrigation evaluation indicators (AE, RE, DU, and Y).

The different scenarios listed in Table 10 were simulated using the WinSRFR5.1 model to study the impact of C_f_ on the irrigation evaluation indicators under different conditions. The simulation results are shown in Fig. 1.

Fig. 1 shows that when C_f_ varies between 0.8 and 1.0, the actual irrigation quota is lower than D_req_, there is no deep percolation loss, and the irrigation efficiency AE is high and changes slowly. The overall trend of DU with C_f_ first increases and then decreases; the results for the different design schemes vary greatly. With increasing D_req_, the peak value of DU shifts to the right. This shows that the higher D_req_ is, the lower q and the longer the irrigation time for achieving a uniform irrigation distribution. Contrary to the trend of AE, RE increases with increasing C_f_; namely, the water storage capacity of the crop root layer increases with increasing irrigation amount and finally reaches 1.00. All the water stored in the crop root zone is irrigation water. With increasing C_f_, the overall comprehensive indicator Y also shows a trend of first increasing and then decreasing. The Y value is the largest at the inflection point of the curve, and the inflection point is the best C_f_ under D_req_ and q.

When D_req_ is 60 mm and q is 7.0 L s^−1^ m^−1^, C_f_ at the inflection point of the comprehensive index curve Y is 1.00; when D_req_ is 80 mm and q is 5.2 L s^−1^ m^−1^, C_f_ at the inflection point is 1.06; and when D_req_ is 100 mm and q is 3.8 L s^−1^ m^−1^, C_f_ at the inflection point is approximately 1.10. This matches the conventional design results for the border irrigation variables, which indirectly verifies the accuracy of the results.

### 3.3 Effect of C_f_ on the loss functions

**Fig. 2:**
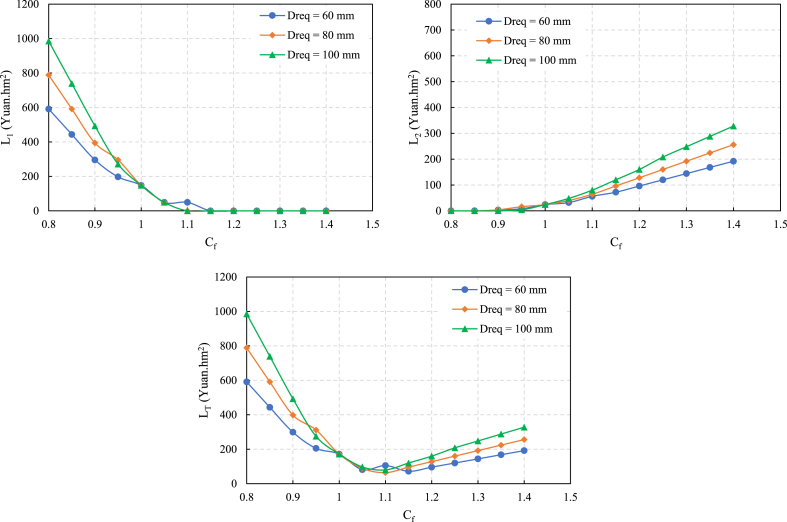
Influence of Cf on the loss functions (L1, L2, and LT).

The impact of C_f_ on the three types of losses is shown in Fig. 2.

The crop yield loss L_1_ gradually decreases to zero with increasing C_f_; the irrigation water gradually increases to meet the crop demand fully. The irrigation water loss L_2_ exhibits the opposite. With increasing C_f_, L_2_ gradually increases, and the water loss caused by excessive irrigation increases. From a quantitative comparison perspective, since the water fee in the experimental area is lower than the wheat purchase price, L_2_ associated with overirrigation is much lower than the L_1_ associated with underirrigation. L_T_ exhibits an upward unimodal parabolic shape, and with increasing D_req_, L_T_ becomes increasingly significant and less likely to decrease. Therefore, when the C_f_ value diverges from the parabola's nadir, there will be a corresponding increase in the LT value. Consequently, ensuring that the actual irrigation level closely aligns with the design level will notably enhance the overall design. In Fig. 2, C_f_ corresponding to the bottom of the parabola is the correction coefficient results in L_T_ minimization under D_req_ and q.

Under the conditions of the three design irrigation quotas D_req_, the C_f_ at the minimum L_T_ value is 1.10 for D_req_ values of 80 mm and 100 mm, and 1.15 for a D_req_ of 60 mm, which is slightly larger than the conventional design value. The results show that from the perspective of the loss functions, L_T_ of the conventional design results based on the irrigation evaluation indicators as the evaluation target is not the minimum. Based on the conventional design results, further increasing C_f_ could induce more minor economic losses.

### 3.4 Optimization of the irrigation variables based on the loss model

The underirrigation loss and overirrigation loss caused by uneven irrigation can be converted into the economic loss, which is the total loss L_T_ associated with uneven irrigation. Starting from the perspective of the economic losses caused by uneven border irrigation, considering L_T_ minimization as the objective function and combining uniform design theory and the WinSRFR5.1 model, a loss model for uneven border irrigation was established to optimize the design of the irrigation variables (q and C_f_).

To minimize the loss of economic benefits due to uneven border irrigation, the minimum L_T_ value is set as the goal, as follows:

$$f=\min\;L_{T}=\min\left(L_{1}+L_{2}\right)$$

The constraints of the loss model include q and C_f_. According to the results in the previous sections, the constraints for determining the loss model are as follows:

$$\left\{ \begin{array}{c} 3.0\;L/\left(m.s\right)\leq q\leq 9.0\;L/\left(m.s\right)\\ 0.80\leq C_{f}\leq 1.38 \end{array}\right.$$

The uniform design table U30∗(30^2^) was applied to the tested scenarios, under the constraints of the decision variables, to select different combinations of q and C_f_. Then, the WinSRFR5.1 model was used to simulate and obtain infiltration water distribution results under each scenario. The other parameters fed into the WinSRFR model are similar to those in the previous sections (k = 122.24 mm.min^-a^, a = 0.68, slope = 0.0025, Manning's coefficient = 0.06, L = 100 m, W = 3.7 m, and D_req_ = 60 mm).

The total loss L_T_ under each scenario was determined, and the results were analyzed using regression equations. The scenario with the lowest L_T_ value is the optimal design scenario.

The goal is to improve the water use efficiency and obtain higher crop yields. The optimization of irrigation variables based on the border irrigation loss model can ensure suitable irrigation evaluation indicators and minimize the total economic losses associated with uneven irrigation. The uniform design table U30∗(30^2^)-based values of q and C_f_ and the loss model calculation results are shown in Table 11.

**Table 11:** Optimization design results for the irrigation variables (D_req_ = 60 mm).

Level	q L.s^−1^.m^−1^	C_f_	Q L.s^−1^	tco min	L_1_ yuan.hm^−2^	L_2_ yuan.hm^−2^	L_T_ yuan.hm^−2^
1	7.6	1.28	28.1	16.8	0.00	136	136.00
2	7.4	0.90	27.4	12.2	246.40	8	254.40
3^a^	3.0	1.02	11.1	34.0	0.00		
4	4.6	1.14	17.0	24.8	98.56	80	178.56
5^a^	3.8	1.10	14.1	28.9	0.00		
6^a^	5.0	0.92	18.5	18.4	0.00		
7	6.6	0.86	24.4	13.0	443.52	0	443.52
8^a^	4.0	0.96	14.8	24.0	0.00		
9	7.0	1.00	25.9	14.3	147.84	24	171.84
10^a^	3.4	0.84	12.6	24.7	0.00		
11	5.2	1.06	19.2	20.4	49.28	40	89.28
12	5.8	1.18	21.5	20.3	0.00	88	88.00
13	6.8	1.38	25.2	20.3	0.00	184	184.00
14	6.4	1.08	23.7	16.9	49.28	48	97.28
15	6.2	1.26	22.9	20.3	0.00	120	120.00
16	8.0	0.82	29.6	10.3	537.15	0.8	537.95
17	4.2	1.36	15.5	32.4	49.28	176	225.28
18^a^	3.6	1.20	13.3	33.3	0.00		
19	8.4	1.04	31.1	12.4	147.84	48	195.84
20	8.2	1.34	30.3	16.3	0.00	160	160.00
21	7.8	1.12	28.9	14.4	49.28	64	113.28
22	5.4	1.32	20.0	24.4	0.00	152	152.00
23^a^	5.6	0.80	20.7	14.3	0.00		
24	8.6	0.94	31.8	10.9	344.96	24	368.96
25	7.2	1.16	26.6	16.1	49.28	80	129.28
26^a^	4.4	0.88	16.3	20.0	0.00		
27^a^	3.2	1.30	11.8	40.6	0.00		
28	6.0	0.98	22.2	16.3	98.56	8	106.56
29	8.8	1.22	32.6	13.9	49.28	112	161.28
30	4.8	1.24	17.8	25.8	49.28	120	169.28

a The advance phase was not completed, and these levels were not considered in the following analysis.

The significance test of the optimal design of the second-order equation regression is as follows: the second- and third-order regression equations are still used to analyze the results in Table 11.

The results of the second-order equation regression are as follows: the regression results show that both the second-order regression equation and its regression coefficient meet the highly significant correlation requirements (Table 12, Table 13).

**Table 12:** Significance test of the second-order regression equation (D_req_ = 60 mm).

Multiple R	R^2^	Adjusted R^2^	Standard Error	*F*	*Significance F*
0.943	0.981	0.863	43.77	32.5	1.68E-7

**Table 13:** Second-order regression equation coefficients and test values (D_req_ = 60 mm).

Variable Name	*Coefficients*	*Standard Error*	*T Stat*	*P Value*
Intercept	3370.41	529.68	6.36	9.4095E-06
C_f_	−5607.19	943.15	−5.95	2.0548E-05
q^2^	10.41	3.06	3.40	0.0036717
C_f_^2^	2686.47	383.66	7.00	2.9841E-06
qXC_f_	−110.58	34.10	−3.24	0.00510062

Notably, there is no q variable in the second-order regression equation in Table 12 because q is not significant (P > 0.05) and is eliminated by the backward elimination method.

The GA was used to obtain the minimum value of the second-order regression equation. The optimal combination of the irrigation variables for a design irrigation quota D_req_ of winter wheat of 60 mm was as follows: q = 6.22 L s^−1^ m^−1^, and C_f_ = 1.17.

The third-order regression significance test results are listed in Table 14, Table 15. Both the third-order regression equation and its regression coefficient meet the extremely significant correlation requirements.

**Table 14:** Significance test of the third-order regression equation (D_req_ = 60 mm).

Multiple R	R^2^	Adjusted R^2^	Standard Error	*F*	*Significance F*
0.937	0.878	0.847	46.28	28.67	4.04E-7

**Table 15:** Third-order regression equation coefficients and test values (D_req_ = 60 mm).

Variable Name	*Coefficients*	*Standard Error*	*T Stat*	*P Value*
Intercept	2512.03	374.89	6.70	5.09E-06
C_f_	−3087.78	500.99	−6.16	1.36E-05
q^3^	0.54	0.17	3.23	0.0052
C_f_^3^	904.15	117.96	7.66	9.62E-07
qXC_f_^2^	−46.43	15.81	−2.94	0.0097

The GA was used to calculate the minimum value of the above third-order regression equation, and the optimal combination of the irrigation variables for a design irrigation quota D_req_ of winter wheat of 60 mm was q = 6.30 L s^−1^ m^−1^, and C_f_ = 1.18.

Similar to the conventional design regression optimization results, the optimal design results show that the results of the second-order regression equation differ from those of the third-order regression equation. To ensure that the best solution is accurate and reasonable, the results of the second- and third-order regression equations were fed into the WinSRFR3.1 model, and the corresponding economic losses were calculated; the results were compared with the test scenarios (Table 11). Finally, the scenario with the lowest total economic loss was selected. The results are shown in Table 16.

**Table 16:** Selection of the best solution for the recommended design (D_req_ = 60 mm).

Method	q L.s^−1^.m^−1^	C_f_	AE	DU	RE	Y	L_1_ yuan.hm^−2^	L_2_ yuan.hm^−2^	L_T_ yuan.hm^−2^
Level 12	5.80	1.18	0.85	0.89	1	0.911	0	88	88
Second Order	6.22	1.17	0.86	0.89	1	0.915	0	80	80
Third Order	6.30	1.18	0.85	0.89	1	0.911	0	88	88

For D_req_ = 60 mm, the best level for the border irrigation variables is the second-order regression equation, and the total loss is 80 yuan.hm^−2^. The irrigation evaluation indicators are AE = 0.86, DU = 0.89, RE = 1.00, and Y = 0.915.

Under winter wheat D_req_ variation, the optimization results for the border irrigation variables are shown in Table 17. With increasing D_req_, q decreases, and C_f_ increases. The optimized design results under the three design irrigation quotas D_req_ yield the lowest total loss and relatively high irrigation evaluation indicators, indicating that the optimal design scheme provides better irrigation effects.

**Table 17:** Recommended design results for the different water requirement quotas.

D_req_ mm	q L.s^−1^.m^−1^	C_f_	AE	DU	RE	Y	L_1_ yuan.hm^−2^	L_2_ yuan.hm^−2^	L_T_ yuan.hm^−2^
60	6.22	1.17	0.86	0.89	1.00	0.915	0.00	80	80.00
80	4.60	1.14	0.88	0.90	1.00	0.930	1.23	88	89.23
100	3.80	1.10	0.91	0.91	1.00	0.940	0.00	80	80.00

The irrigation plan based on the optimization calculation of the loss model allows most border fields to reach the designed irrigation quota D_req_; that is, the irrigation amount of most border fields meets the crop growth requirements. At the same time, the optimization plan also minimizes the loss of irrigation volume caused by uneven border irrigation.

### 3.5 Comparison of the optimal design with the conventional design

The conventional approach prioritizes the maximum comprehensive evaluation indicator Y as the objective function, whereas the optimal design focuses on the minimum economic loss L_T_ as the objective function. This distinction carries significant implications for practical irrigation management and resource conservation. When aiming to minimize L_T_, the emphasis shifts towards achieving high crop yields and reducing water loss. In contrast, maximizing Y may enhance water use efficiency but not necessarily crop yield. Thus, optimizing design provides a more comprehensive understanding of irrigation variables and their impact on crop yield and water losses.

**Fig. 3:**
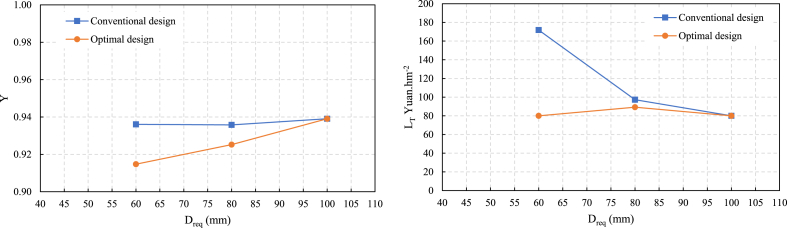
Comparison of Dreq and (a) evaluation indicators Y and (b) LT between the conventional and optimal designs.

The Y and L_T_ results of the two design methods are shown in Fig. 3. When the Dreq ranges from 60 to 80 mm, the Y and L_T_ values under the optimal design results are lower than those under the conventional design. This suggests improved efficiency and resource utilization in the optimal design scenario. Additionally, the increase in C_f_ in the optimization results corresponds to a higher actual irrigation quota (m). While this may lead to increased irrigation water loss L_2_ due to overirrigation, it also significantly reduces crop yield loss L_1_ associated with underirrigation.

Notably, crop yield loss L_1_ can decrease to zero under optimal design conditions in specific scenarios (i.e., when D_req_ is 60 mm, L_1_ decreases from 147 yuan.hm^−2^ under conventional design (Table 11, level 9) to zero yuan.hm^−2^ under optimal design (Table 17).), and the total loss L_T_ decreases.

**Fig. 4:**
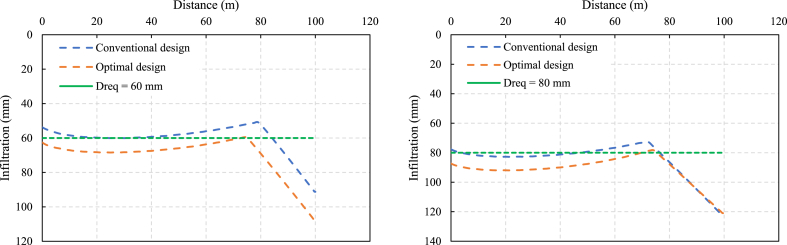
The infiltration depth for the conventional and optimal design.

The optimal and conventional design results show that with increasing D_req_ from 60 to 80 mm, q decreases by approximately 43 %, accompanied by an increase in tco (Fig. 4). This reduction in q in the optimization results signifies improved water conservation efforts. Under the same design irrigation quota, when D_req_ is between 60 and 80 mm, q in the optimization results is 15 % lower than in conventional results. C_f_ in the optimization results is more significant than in the conventional results. This mainly occurs because of the higher loss value associated with underirrigation L_1_ than overirrigation L_2_.

**Fig. 5:**
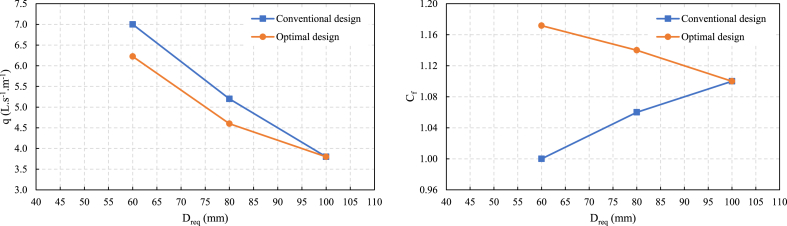
Comparison of D_req_ and (a) inflow rate q and (b) C_f_ between the conventional and optimal designs.

Within a reasonable range, increasing the actual irrigation quota causes a reduction in the economic losses caused by uneven border irrigation. However, when D_req_ reaches 100 mm, the results of both design methods converge, as a higher design irrigation quota D_req_ reduces the impact of the underirrigation loss. A larger D_req_ leads to longer irrigation time for the same inflow rate value, effectively decreasing the deficit area and mitigating underirrigation losses. These insights highlight the intricate relationship between design parameters, irrigation efficiency, and resource conservation, emphasizing the importance of optimizing irrigation systems for sustainable agricultural practices. A comparison of the results of the two design methods is shown in Fig. 5.

## 4 Conclusion

In this study, the irrigation quota correction coefficient C_f_ is proposed and applied in the design of border irrigation variables. Based on the loss functions, a total loss model for uneven border irrigation was established to obtain a more reasonable border irrigation design. The main conclusions are as follows.

(1) C_f_ can be used to design border irrigation variables instead of the irrigation time, as C_f_ considers different irrigation quotas. C_f_ has a precise meaning and exerts favorable application effect on the irrigation performance evaluation indexes.

With increasing C_f_, tco increases, and the amount of water entering the border increases, resulting in a decrease in AE. DU shows an initial increase and a subsequent decrease; RE gradually increases and finally reaches 1.00; all irrigation water is stored in the crop root zone. The overall comprehensive indicator Y also shows a trend of first increasing and then decreasing. With increasing C_f_, the crop yield loss value L_1_ gradually decreases to zero; the irrigation water loss value L_2_ indicates the opposite trend, gradually increasing; and the total loss L_T_ exhibits an upward unimodal parabolic shape. C_f_ corresponding to the valley bottom is the correction coefficient that results in L_T_ minimization under the design irrigation quota D_req_ and q. In this study, based on the conventional design results, further increasing C_f_ can yield lower economic losses.

(2) When comparing conventional and optimal design, it was observed that when D_req_ values were between 60 and 80 mm, the Y and L_T_ values achieved through optimal design were 2 % and 50 mm. ha^−2^ lower, respectively, than those obtained through conventional design. Furthermore, the optimization results showed a 15 % decrease in q compared to the conventional results. However, when Dreq was 100 mm, both methods produced similar results.
(3) The controllable irrigation variables of typical winter wheat border fields are optimized based on the total loss model for uneven border irrigation.

The optimal irrigation variables q and C_f_ for design irrigation quotas of 60, 80, and 100 mm are q = 6.22 L s^−1^ m^−1^ and C_f_ = 1.17; q = 4.60 L. s^−1^. m^−1^ and C_f_ = 1.14; and q = 3.80 L s^−1^ m^−1^ and C_f_ = 1.10, respectively. Compared with the conventional design results, the inflow rate per unit width q of the optimal design results based on the total loss model decreases, C_f_ increases, comprehensive indicator Y slightly decreases, and L_T_ significantly decreases.

This study validates the total loss model for a specific soil texture and crop type across a wide range of inflow rates. However, further research is needed to ensure this model is applicable to different irrigation conditions and crop types. Therefore, it is recommended that more diverse testing scenarios be conducted to validate the total loss model thoroughly.

## CRediT authorship contribution statement

**Mohamed Khaled Salahou:** Writing – review & editing, Writing – original draft, Supervision, Software, Methodology, Formal analysis, Data curation, Conceptualization. **Xiaoyuan Chen:** Writing – review & editing, Funding acquisition. **Yupeng Zhang:** Writing – review & editing. **Haishen Lü:** Writing – review & editing. **Xiyun Jiao:** Writing – review & editing.

## Data availability statement

All data used during the study appear in the submitted article.

## Declaration of competing interest

The authors declare that they have no known competing financial interests or personal relationships that could have appeared to influence the work reported in this paper.
